# Liraglutide activates autophagy *via* GLP-1R to improve functional recovery after spinal cord injury

**DOI:** 10.18632/oncotarget.20791

**Published:** 2017-09-08

**Authors:** Jian Chen, Zhouguang Wang, Yuqin Mao, Zengming Zheng, Yu Chen, Sinan Khor, Kesi Shi, Zili He, Jiawei Li, Fanghua Gong, Yanlong Liu, Aiping Hu, Jian Xiao, Xiangyang Wang

**Affiliations:** ^1^ Department of Orthopaedic Surgery, The Second Affiliated Hospital and Yuying Children’s Hospital of Wenzhou Medical University, Wenzhou, China; ^2^ Molecular Pharmacology Research Center, School of Pharmaceutical Sciences, Wenzhou Medical University, Wenzhou, Zhejiang, China; ^3^ Department of Molecular Pharmacology, Albert Einstein College of Medicine, Bronx, NY, U.S.A

**Keywords:** spinal cord injury, apoptosis, liraglutide, GLP-1R, autophagy

## Abstract

Therapeutics used to treat central nervous system (CNS) injury are designed to promote axonal regeneration and inhibit cell death. Previous studies have shown that liraglutide exerts potent neuroprotective effects after brain injury. However, little is known if liraglutide treatment has neuroprotective effects after spinal cord injury (SCI). This study explores the neuroprotective effects of liraglutide and associated underlying mechanisms. Our results showed that liraglutide could improve recovery after injury by decreasing apoptosis as well as increasing microtubulin acetylation, and autophagy. Autophagy inhibition with 3-methyladenine (3-MA) partially reversed the preservation of spinal cord tissue and decreased microtubule acetylation and polymerization. Additionally, siRNA knockdown of GLP-1R suppressed autophagy and reversed mTOR inhibition induced by liraglutide *in vitro*, indicating that GLP-1R regulates autophagic flux. GLP-1R knockdown ameliorated the mTOR inhibition and autophagy induction seen with liraglutide treatment in PC12 cells under H_2_O_2_ stimulation. Taken together, our study demonstrated that liraglutide could reduce apoptosis, improve functional recovery, and increase microtubule acetylation via autophagy stimulation after SCI. GLP-1R was associated with both the induction of autophagy and suppression of apoptosis in neuronal cultures.

## INTRODUCTION

Acute traumatic spinal cord injury (SCI) is a devastating disease that causes severe neurological deficits. The pathological process of SCI can be divided into two distinct stages: the primary injury, caused by a direct, local, and segmental damage at the spinal cord; and the secondary injury, which includes a complex cascade of molecular events including inflammatory responses, local edema, posttraumatic ischemia, hemorrhage, excitotoxicity, and axonal disruption [1, 2]. The neurotoxicity incited by the secondary phase of injury leads to neuronal death and destruction of neurocircuitry, which is one of the major reasons for the physical and functional deficits seen after SCI [3]. Thus, therapies to treat SCI or CNS injury are targeted at inhibiting apoptosis and maintaining functional neural circuitry [3, 4].

Axon regeneration plays a critical role in the treatment of CNS injury [5, 6]. Neurons have highly polarized structures including one hair-like extension that transmits signal (the axon) and several tapered, shorter extensions that receive signal (the dendrites) [7]. Cytoskeletal remodeling, such as the assembly of microtubules, is demonstrated to be pivotal for growth cone initiation and subsequent repair of injured axons [8, 9]. Previous studies have reported that microtubule dynamics are increased by nascent microtubule nucleation through JNK signaling. Upregulating microtubule dynamics attenuate acute injury-induced axonal degeneration, counteract progressive longer-term degeneration seen in poly-Q proteins. Thus, acute axonal injury and chronic neuronal stress induce a cytoskeleton-based stabilization process [10]. In recent years, pharmacological treatment to stabilize microtubulin was capable of promoting axon regeneration after SCI [11]. Additionally, it was reported that epothilone B could reactivate polarization of neuronal structure via coordinated microtubule polymerization at the distal end of the axon, propelling axonal regeneration in an inhibitory environment [12].

Axon regeneration involves extensive remodeling of cytoplasmic compartments and axonal structures through the synthesis and degradation of local proteins [13]. Autophagy, derived from the Greek phrase meaning “self-eating”, is an intracellular catabolic process of cytoplasmic compositions in the autophagosomal pathway [14]. In the nervous system, autophagosomes have been observed in cultured neurons and *in vivo* [15]. Crush lesion on the optic nerve triggers a rapid calcium influx into neurons followed by autophagy induction, which leads to axon degradation [16]. Interestingly, administration of Tat-beclin1 stabilized microtubules and promoted axon regeneration after SCI [8]. These results indicate that modulation of autophagy activity may play a critical role in axon regeneration after CNS injury.

Glucagon-like peptide 1 (GLP-1) is a gut-derived incretin hormone, which is secreted into the blood stream and binds to the GLP-1 receptor (GLP-1R). It is best characterized by its role in blood glucose homeostasis [17]. However, GLP-1 and its longer-lasting analog, Ex4, may have multiple synergistic effects on neural plasticity by protecting against the pyridoxine induced-sensory peripheral neuropathy [18]. Moreover, the distribution of GLP-1R in the brain indicates that they protect brain tissue and neuronal activity. Increasing evidences of cellular and animal neurodegeneration models support the neuroprotective effects of GLP-1R [19, 20]. And the GLP-1 analogs liraglutide, exendin-4, and lixisenatide are approved for treatment of type 2 diabetes mellitus [21, 22]. Liraglutide avoids proteolytic degradation by dipeptidyl peptidase-4 (DDP-4) via combination with serum albumin, extending its half-life compared with other GLP-1 analogs [23]. Liraglutide has also been reported to pass through the blood–brain barrier (BBB), physiologically affecting neurogenesis in rat brains [24]. And ligraglutide had therapeutic effects on metabolic regulation, synaptic plasticity, hippocampal neurogenesis, and cell survival, and promote cognitive function in mice model of obesity with insulin resistance [25]. Exendin-4 could significantly promote locomotion recovery in rats after SCI through the induction of autophagy and inhibition of apoptosis [26].

However, the molecular mechanisms of liraglutide in the nervous system are poorly understood. In present study, we aimed to demonstrate the therapeutic effects of liraglutide on acute SCI both *in vivo* and *in vitro* as well as the relationship between autophagy and microtubule stabilization. We also explored whether mechanism by which liraglutide utilizes the GLP-1R to regulate autophagy, mammalian target of rapamycin (mTOR) signaling, and apoptosis. Liraglutide may prove to be a novel therapeutic intervention to treat SCI as well as other traumatic CNS diseases.

## RESULTS

### Liraglutide ameliorates the damage of spinal cord injury, motor neuron loss and locomotor deficit after SCI *in vivo*

**Figure 1:**
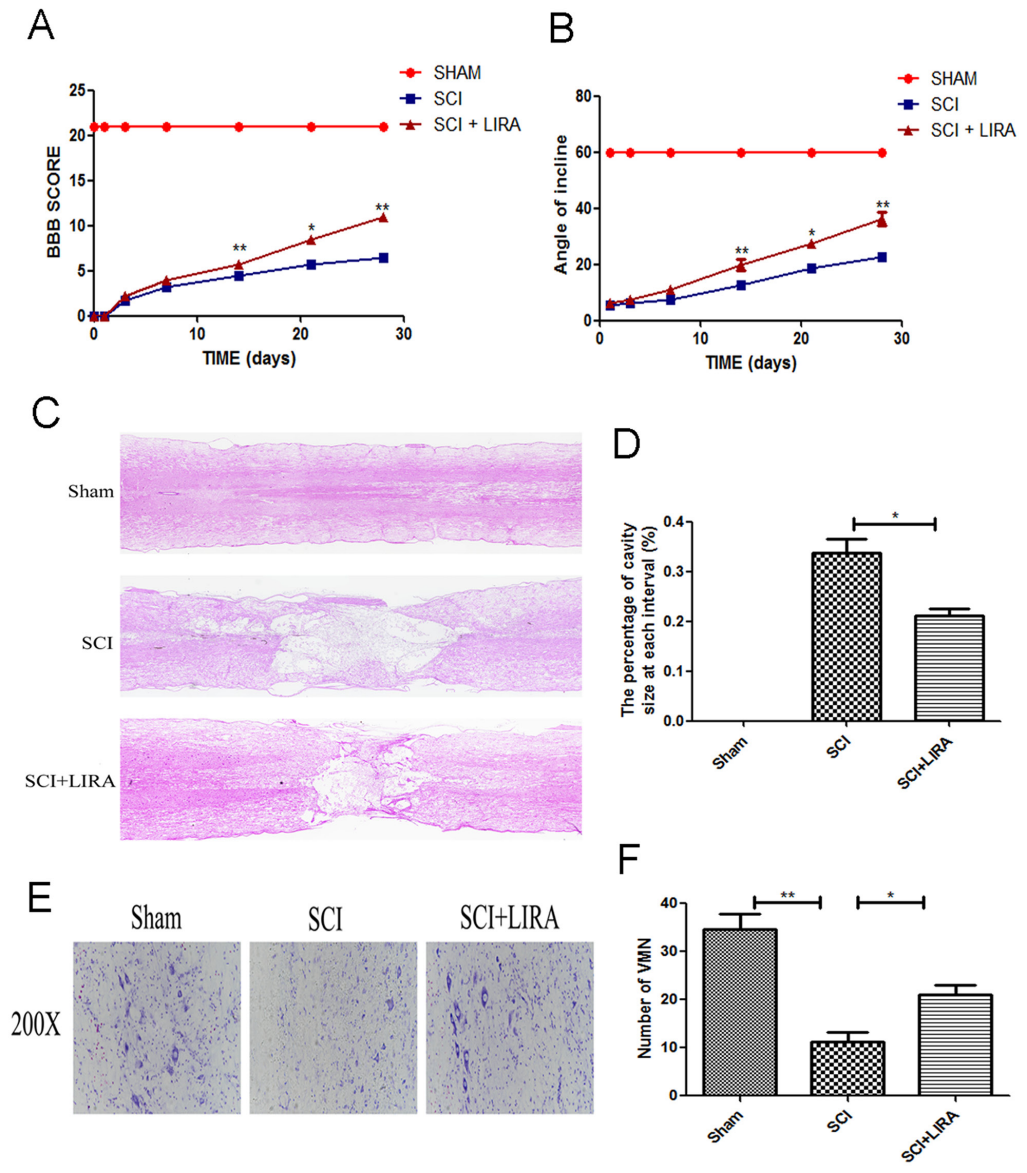
Liraglutide decreases the cavity of necrotic tissue as well as the loss of motor neurons and improves functional recovery after SCI **(A)** The BBB scores of each group, *P < 0.05 versus the SCI group, **P<0.01, n=5. **(B)** The inclined plane test scores of each group, *P<0.05 versus the SCI group, **P<0.01, n=5. **(C-D)** HE staining at 28 days of each group. **(E)** Nissl staining to assess the loss of neurons at 28 days. **(F)** Counting analysis of VMN in each group. Graphic presentation of the percent of cavity of necrotic tissue at each interval; columns represent mean ± SD, *P<0.05 versus the SCI group, n=5.

To evaluate the therapeutic effect of liraglutide on acute traumatic SCI, BBB scores and inclined plane test scores were used to assess locomotor recovery was for 4 weeks by. As shown in Figure 1A, BBB scores after SCI were significantly below the normal score with no significant difference during the first week after surgery. However, compared with the SCI group, BBB scores markedly increased in the liraglutide group at 14, 21, and 28 days after surgery, indicating that locomotor function was significantly improved with the treatment of liraglutde. Similarly, inclined plane test scores were consistent with the BBB scores (Figure 1B). These results indicate that liraglutide may promote functional recovery after SCI. On the HE-stained sections, the cavity of necrotic tissue was delineated with a dashed line. SCI group displayed significant destruction of central gray matter and peripheral white matter. In comparison, the liraglutide-treated group had a decreased cavity of necrotic tissue at the injury site, showing liraglutide protected against more severe damage after SCI (Figure 1C and 1D). The effect of liralutide on surviving motor neurons in the anterior horn was measured by Nissl staining at 28 days after surgery. The SCI group showed remarkable motor neuron loss in the anterior horn, which greatly decreased with liraglutide treatment (Figure 1E and 1F). Thus, liraglutide had a neuroprotective effect on SCI *in vivo*.

### Liraglutide treatment decrease apoptosis in acute SCI

**Figure 2:**
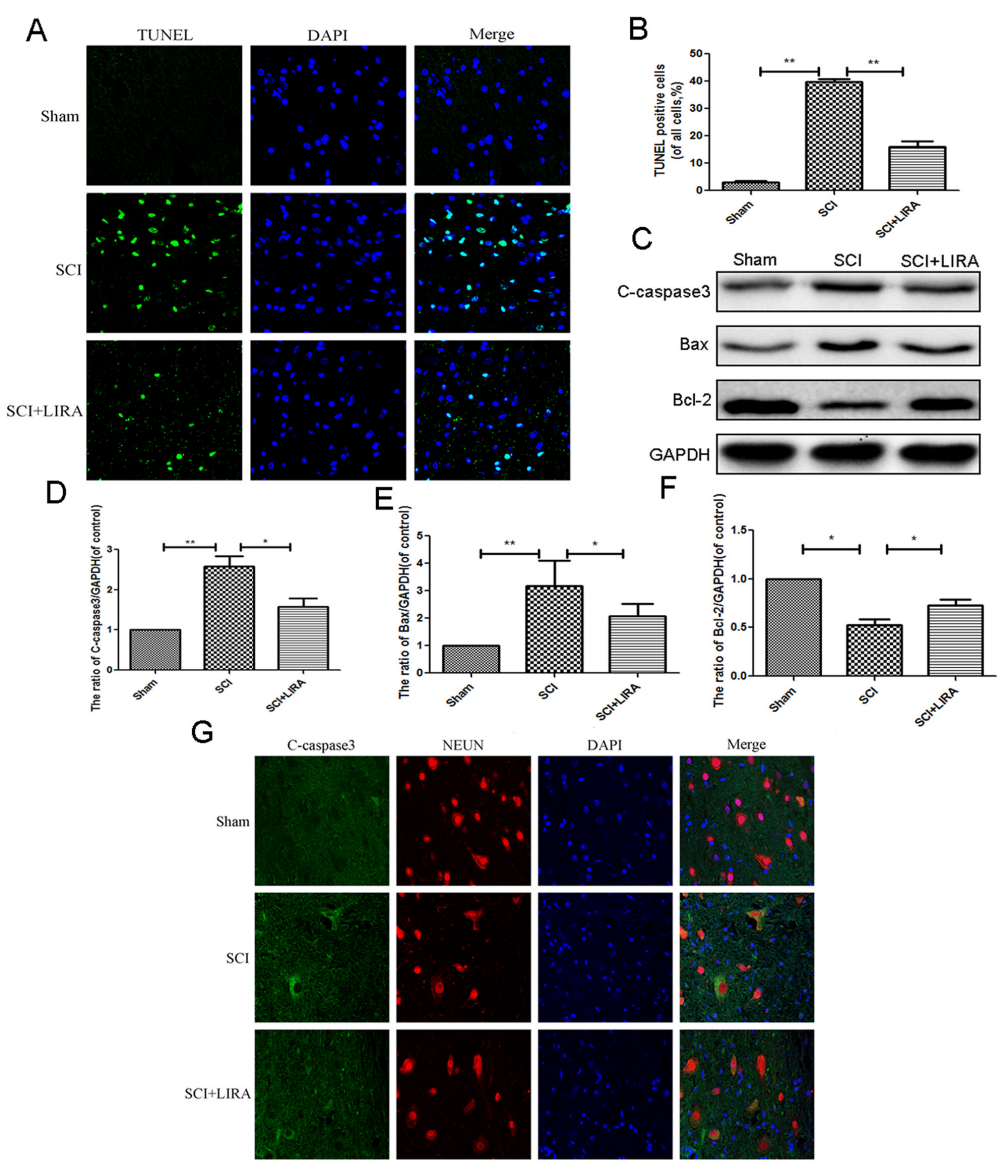
Liragutide reduces apoptosis caused by SCI in rats **(A)** TUNEL staining to assess the apoptosis at 7 days after surgery. **(B)** Quantitative estimation of apoptotic and TUNEL cells. **(C-F)** Representative western blots and quantification data of cleaved caspase 3, Bax and Bcl-2 in each group rats at 3 days after surgery; columns represent mean ± SD, Significant differences between the treatment and sham groups are indicated as *P<0.05, **P<0.01, n=5.

To determine whether liraglutide could decrease apoptosis caused by acute SCI, TUNEL staining was performed to estimate the number of apoptotic cells. Compared with the sham group, SCI group displayed increasing number of apoptotic cells. However, liraglutide treatment greatly reduced apoptotic activity (Figure 2A and 2B). Moreover, Western blot analysis of Bax and cleaved caspase3. Acute SCI increased Bax and cleaved caspase 3 levels, which were significantly reversed with liraglutide treatment. And liraglutide upregulated the anti-apoptotic protein Bcl-2 (Figure 2C-2F). Similarly, immunofluorescent staining showed that caspase3 positive green dots were increased in the SCI group, but reduced with liraglutide treatment (Figure 2G). These results showed that liraglutide could inhibit apoptosis after acute SCI.

### Liraglutide increases microtubule acetylation in acute SCI

**Figure 3:**
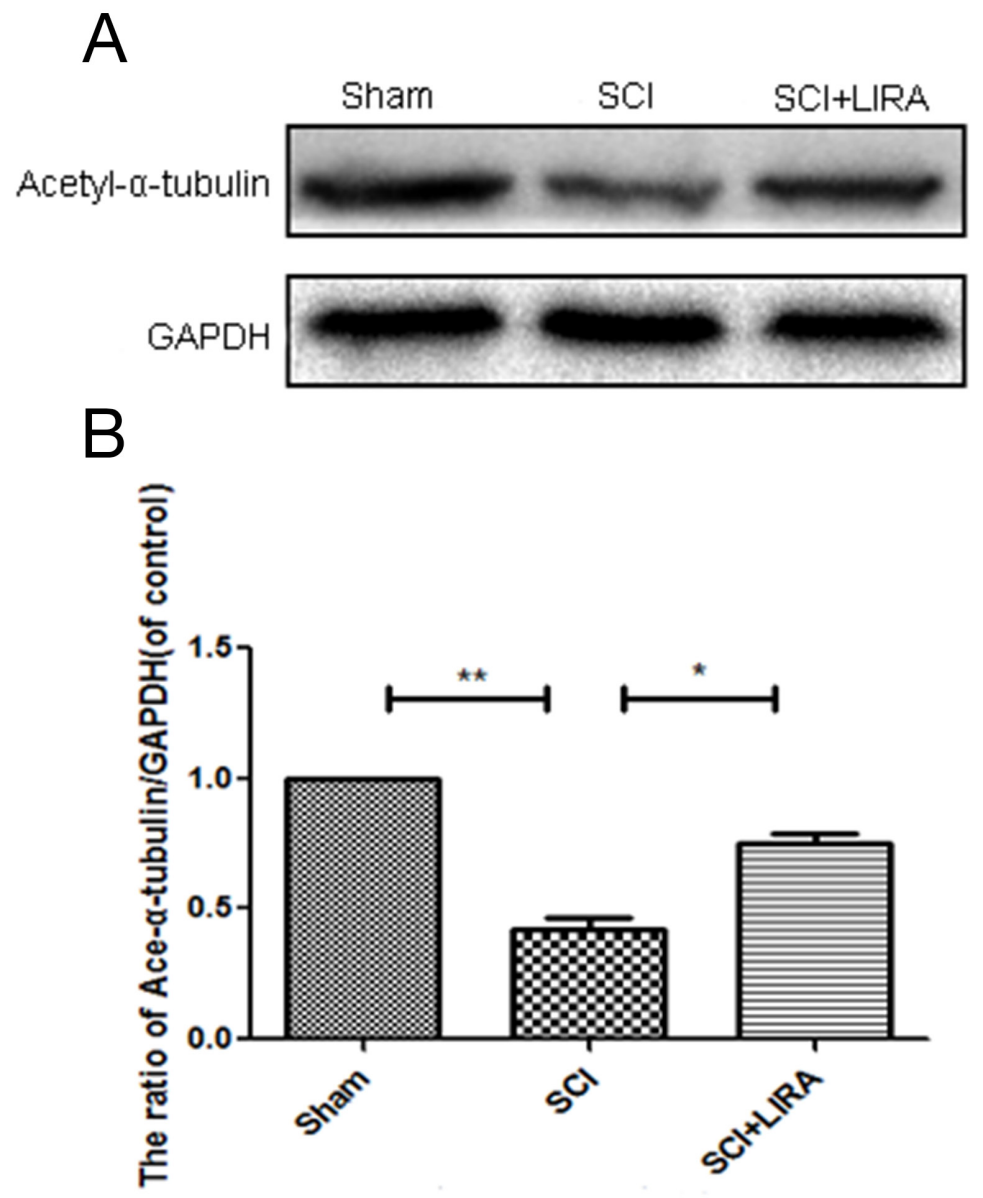
Liraglutide increase microtubule acetylation in acute SCI **(A-B)** Representative western blots and quantification data of acetyl-α-tubulin in each group rats at 28 days after sugery.; columns represent mean ± SD, Significant differences between the treatment and sham groups are indicated as *P<0.05, **P<0.01, n=5.

Acetylation of alpha-tubulin is associated with stable microtubular structures such as axonemes as well as increased axonal transport and reduced locomotor deficits [27, 28]. Acetyl-α-tubulin expression was decreased in the SCI rats compared with the sham. However, liraglutide treatment increased Acetyl-α-tubulin expression after SCI (Figure 3), suggesting that liraglutide may help stabilize microtubules after acute SCI.

### Activation of autophagy is related to liraglutide in the acute SCI

**Figure 4:**
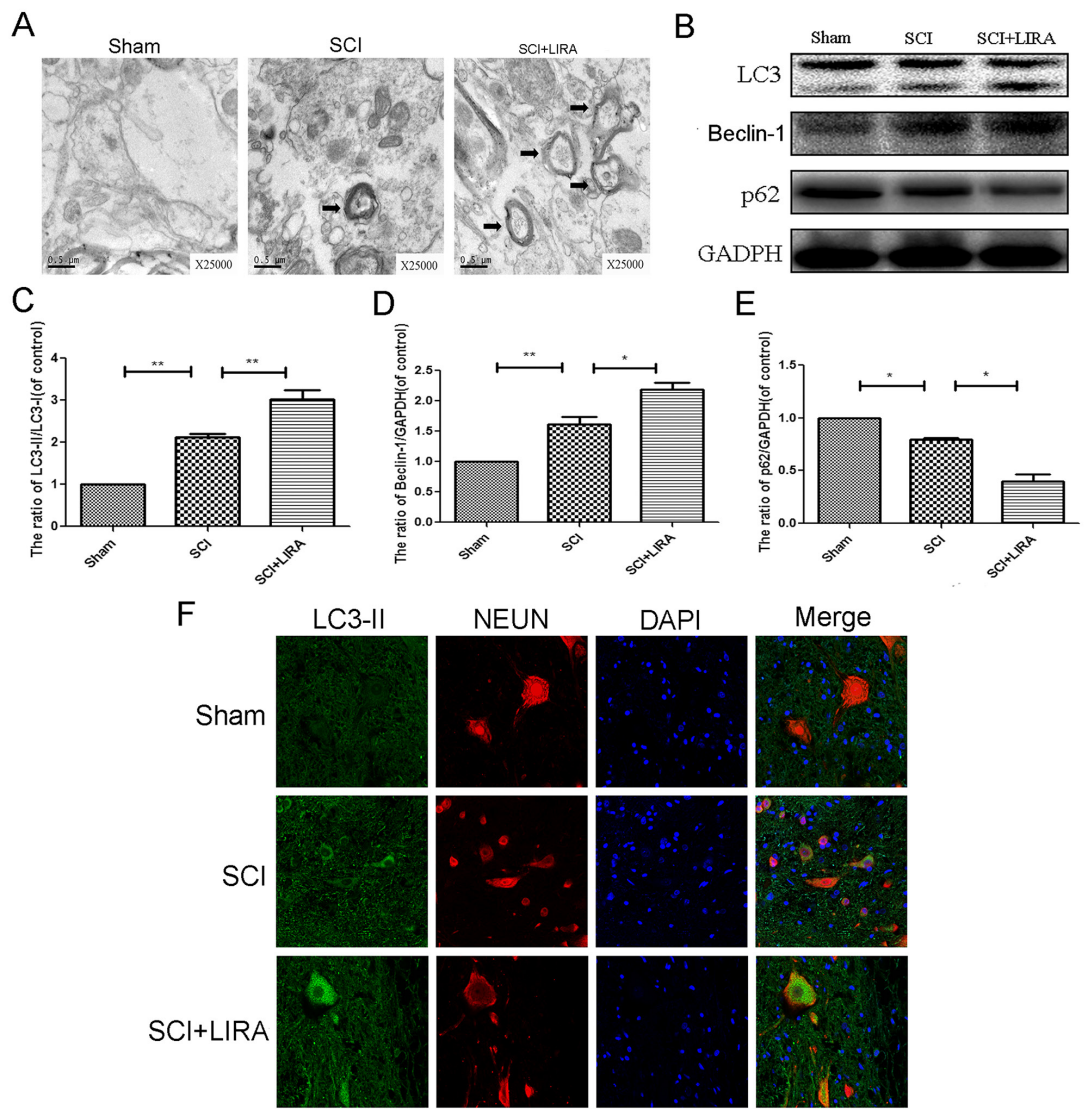
Liraglutide treatment promotes autophagy in rats of SCI **(A)** Transmission electron microscopy showed the autophagosomes (Black arrow: autophagosome) at 3 days after surgery. **(B-E)** Representative western blots and quantification data of LC3, P62 and Benclin-1 protein in each group at 3 days after surgery; columns represent mean ± SD, Significant differences between the treatment and control groups are indicated as **P<0.01, *P<0.05, n=5. **(F)** Double immunofluorescence of NeuN (red) and LC3-II (green) in sections from the injured spinal cord tissue of each group at 3 days after surgery.

While it is known that SCI triggers autophagy activation, the reasoning for this is poorly understood. Using transmission electron microscopy (TEM), we found that increased neuronal autophagosomal vacuoles after SCI and with liraglutide treatment, indicating autophagy activation (Figure 4A). To quantitatively analyze the extent of autophagy induction, we examined known markers of autophagy such as the LC3-II/LC3-I ratio, Beclin-1 and SQSTM1/P62 levels as well as the ratio of p-mTOR/mTOR. As shown in Figure 4B-4E, we found higher ratio of LC3-II/LC3-I and increasing level of beclin-1 in the SCI group compared with the sham group. The ratio of p-mTOR/mTOR and level of p62 decreased, which further supported autophagy activation. However, liraglutide treatment further increased the LC3-II/LC3-I ratio and beclin-1 levels compared with the SCI group. p-mTOR/mTOR ratio and p62 were decreased with liraglutide treatment as well. Similarly, immunofluorescent staining results increased LC3-II positive green dots in the SCI group compared to control sham group, which was exacerbated with liraglutide treatment (Figure 4F).

### Autophagy inhibition abolishes the therapeutic effect of liraglutide treatment on acute SCI

**Figure 5:**
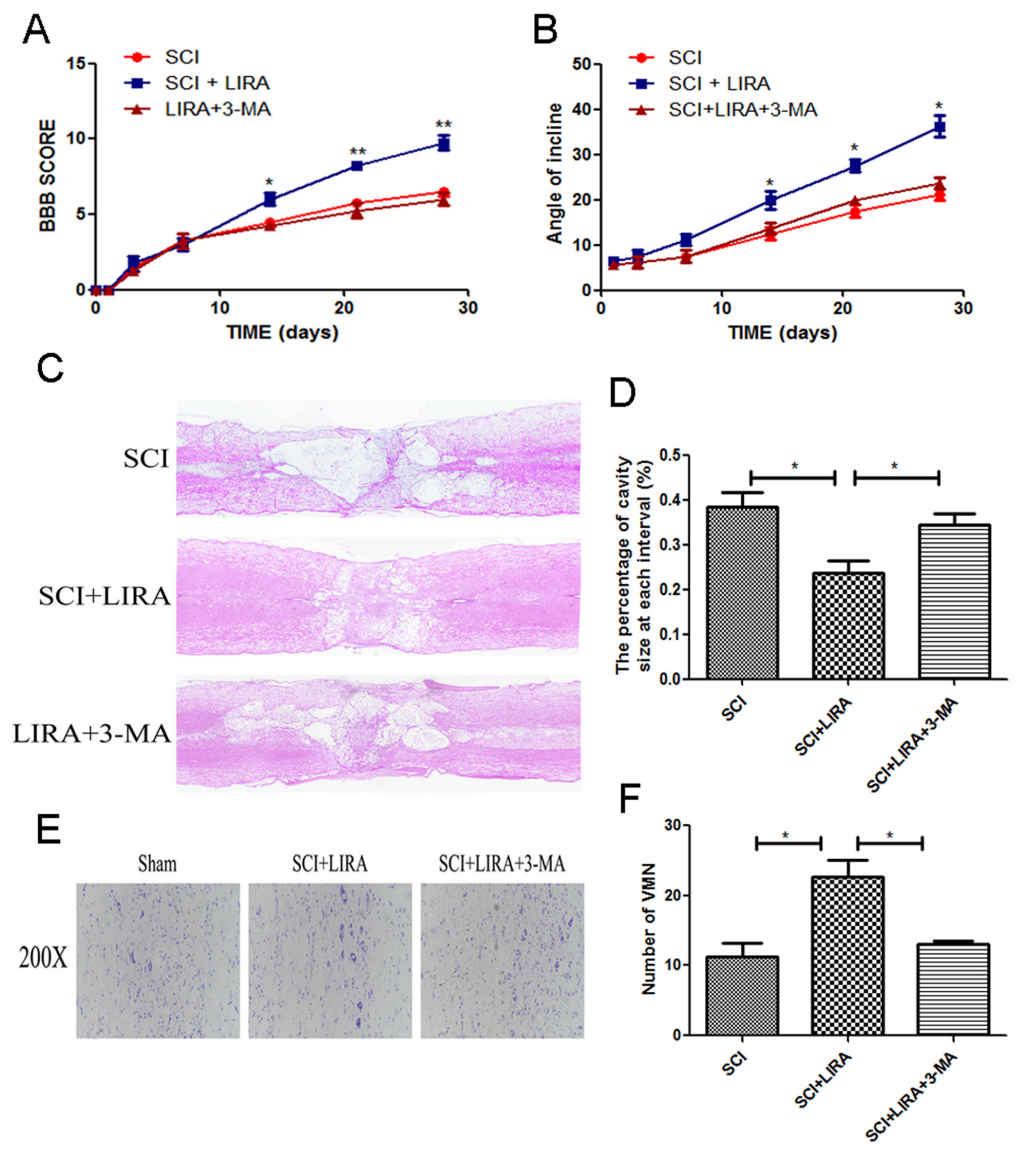
Inhibition of autophagy reversed functional recovery and tissue preservation of liraglutide treatment **(A)** The BBB scores of each group, *P < 0.05 versus the SCI group, **P<0.01, n=5. **(B)** The inclined plane test scores of each group, *P<0.05 versus the SCI group. **(C-D)** HE staining at 28 days of each group. **(E)** Nissl staining to assess the loss of neurons at 28 days after surgery. **(F)** Counting analysis of VMN in each group. Graphic presentation of the percent of cavity of necrotic tissue at each interval; columns represent mean ± SD, Significant differences between the treatment and control groups are indicated as *P<0.05, n=5.

To elucidate whether autophagy was related to SCI recovery improvement seen with liraglutide, we used 3-MA, a classical autophagy inhibitor in conjunction with liraglutide treatment in our SCI model. 3-MA is commonly used to inhibit autophagy *in vivo*. BBB scores and inclined plane test demonstrated that liraglutide’s beneficial effects on locomotor recovery were attenuated by 3-MA (Figure 5A and 5B). 3-MA significantly increased the damage caused by SCI, aggravating the cavity of necrotic tissue compared to liraglutide treatment alone (Figure 5C and 5D). Nissl staining revealed that 3-MA decreased the motor neuron survival in the anterior horn compared to the liraglutide group (Figure 5E and 5F). Taken together, the behavioral tests and histological examination demonstrated that functional autophagy was important for liraglutide’s neuroprotective effect *in vivo*.

### Autophagy inhibition abolished microtubule acetylation and polymerization in acute SCI

**Figure 6:**
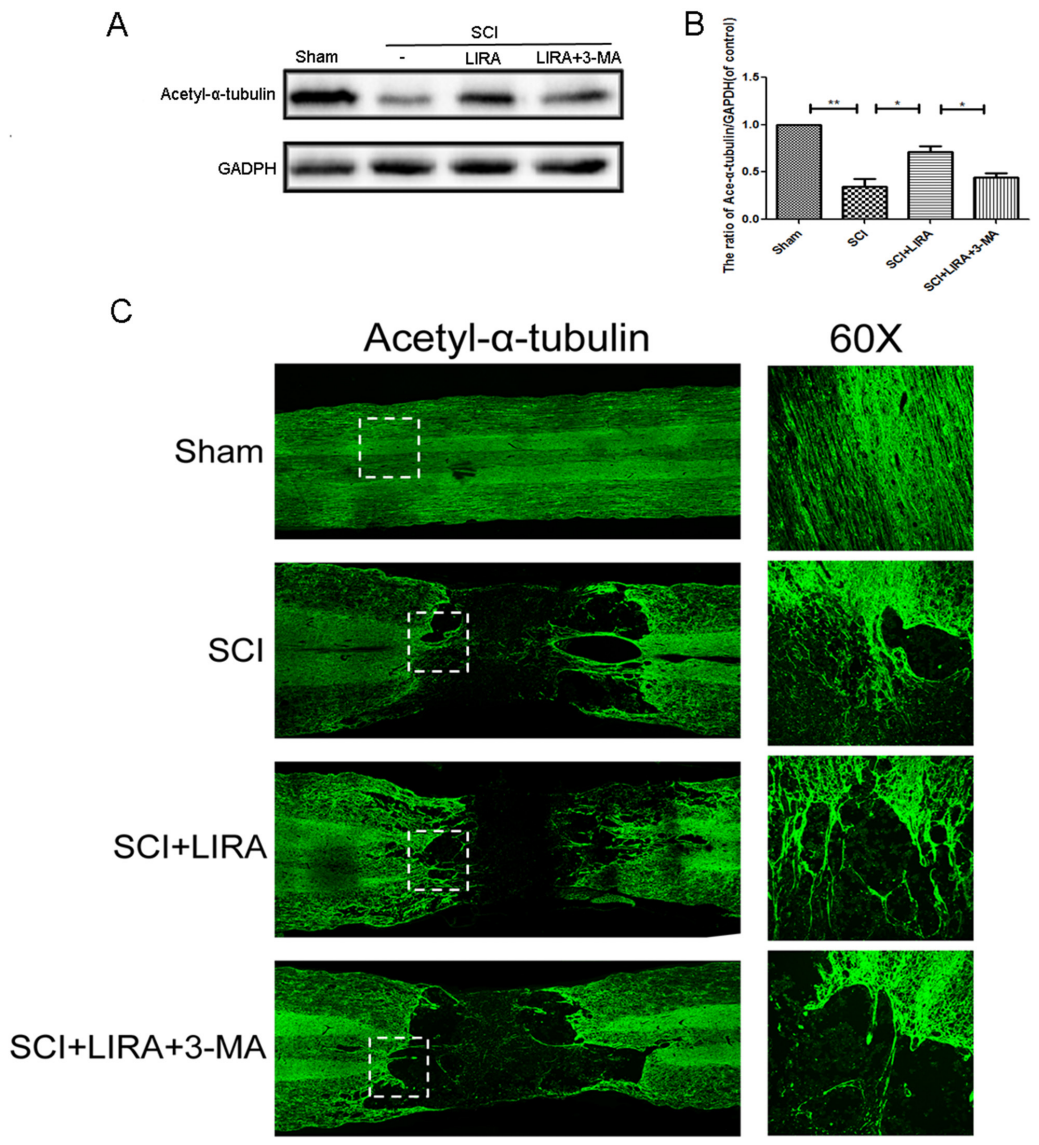
Inhibition of autophagy decreased liraglutide induced microtubule acetylation and microtubule polymerization in acute SCI **(A-B)** Representative western blots and quantification data of acetyl-α-tubulin in each group rats at 28 days after surgery; columns represent mean ± SD, Significant differences between the treatment and sham groups are indicated as *P<0.05, **P<0.01, n=5. **(C)** Immunofluorescence of acetyl-α-tubulin (green) in sections from the injured spinal cord tissue in each group at 28 days after surgery.

To elucidate the role of autophagy in stabilizing neuronal microtubules, we tested acetyl-α-tubulin levels with 3-MA treatment in the liraglutide-treated SCI rats. Western blotting revealed that autophagy inhibition decreased acetyl-α-tubulin compared to liraglutide treatment alone (Figure 6A and 6B). By immunofluorescence, liraglutide treatment promoted microtubule polymerization toward the SCI area and elongation into the distal regions of the SCI area compared to the untreated SCI group. However, 3-MA reversed the liraglutide’s ability to stabilize microtubules (Figure 6C). Thus, liraglutide induced-autophagy increases microtubule stabilization in neurons, indicating that autophagy helps to stabilize microtubules.

### GLP-1R is mainly expressed in spinal cord neurons and significantly increases after acute SCI

**Figure 7:**
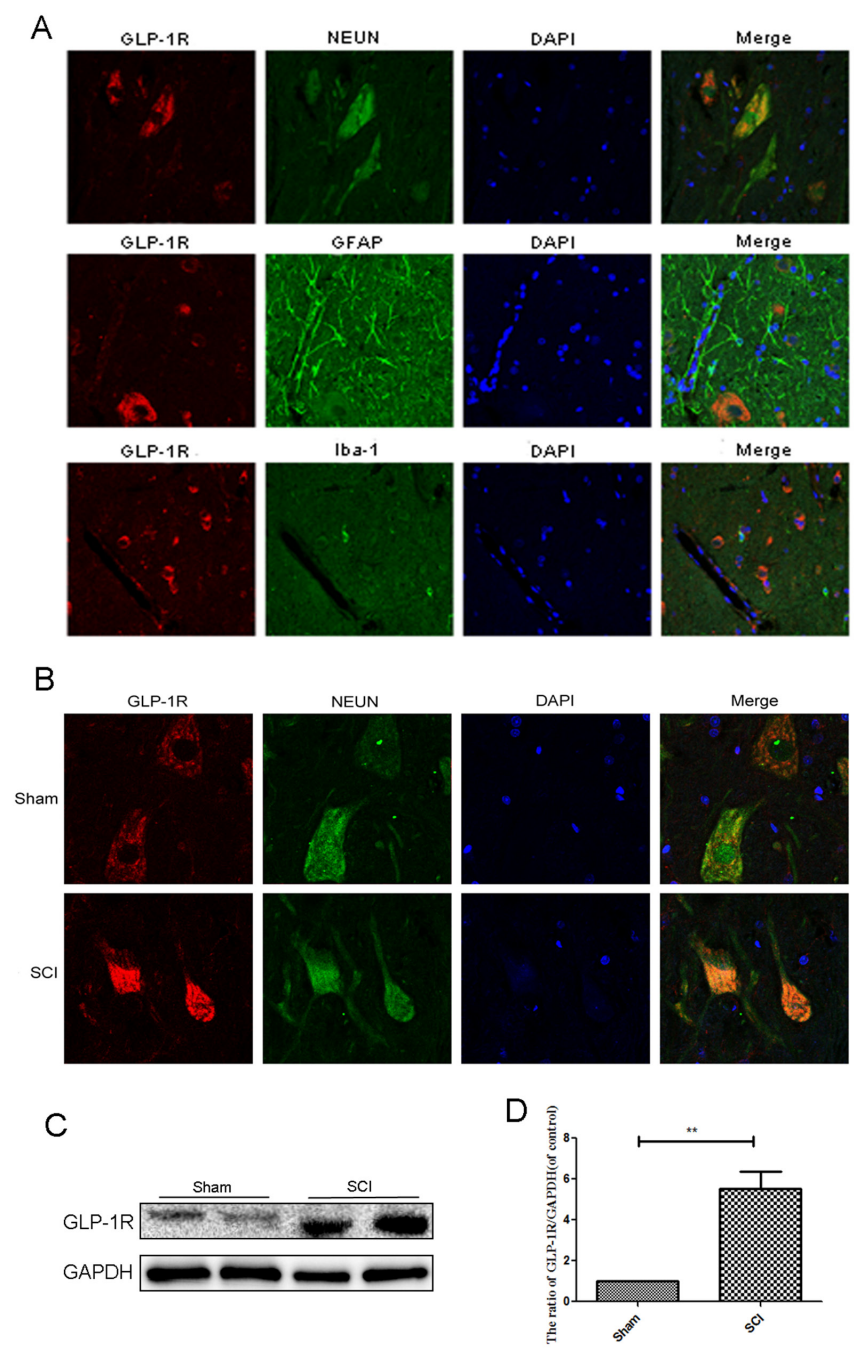
GLP-1R is mainly expressed in neuron in spinal cord tissue and significantly increases after acute SCI **(A)** Double immunofluorescence of GLP-1R (red) and NeuN (neuronal marker, green), GFAP (astrocyte marker, green), Iba-1 (microglia marker, green) in sections from the normal spinal cord tissue. **(B)** Double immunofluorescence of GLP-1R and NeuN in sections from tissue at 1 days after SCI. **(C-D)** Representative western blots and quantification data of GLP-1R in each group rats; columns represent mean ± SD, Significant differences between the SCI and sham groups are indicated as **P<0.01, n=5.

Few articles have reported the expression of GLP-1R in the spinal cord. As shown in Figure 7A, Double immunofluorescence staining for GLP-1R as well as cellular markers for neurons (NEUN), astrocytes (GFAP), and microcytes (Iba-1) showed that GLP-1R is more highly expressed in neurons than astrocytes and microcytes. The SCI group had increased GLP-1R positive puncta in neurons compared with sham group (Figure 7B). Similarly, we observed higher expression of GLP-1R after acute SCI by Western blotting (Figure 7C and 7D).

### GLP-1R knockdown significantly reduced liraglutide-induced autophagy flux and mTOR inhibition in PC12 cells

**Figure 8:**
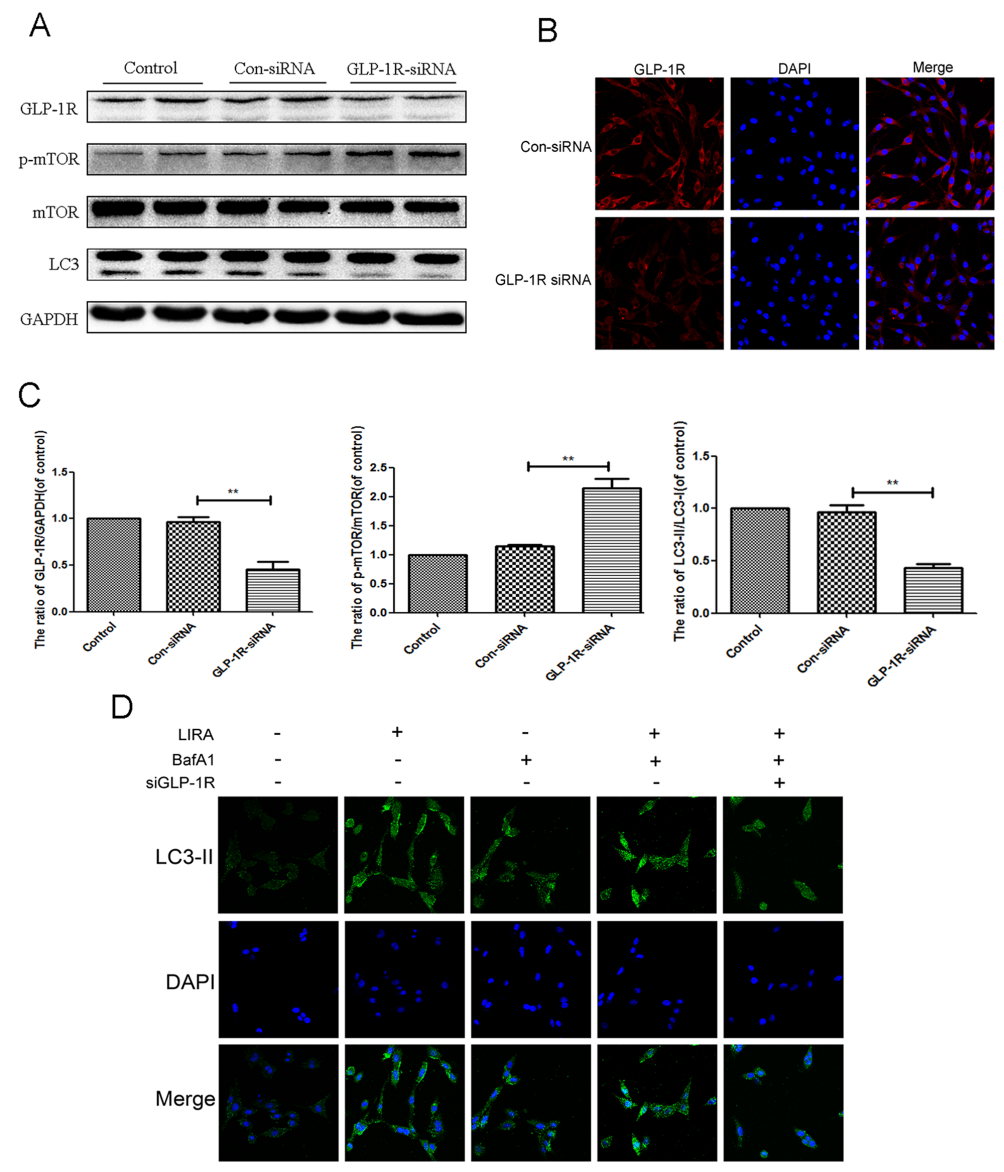
Inhibition of GLP-1R by siRNA markly attenuated liraglutide-induced autophagy in PC12 cells, and reversed the inhibition of mTOR pathway PC12 Cells were transfected with negative control siRNA (con-siRNA) or GLP-1R-siRNA before receiving liraglutide (1 μmol). (**A** and **C-D**) Representative western blots and quantification data of p-mTOR, mTOR and LC3 in the PC12 cells as treated above. Columns represent mean ± SD, significant differences between the treatment and control groups are indicated as **P<0.01, *P<0.05, n=5. **(B)** The GLP-1R (red) was detected by immunofluorescence staining combined with DAPI staining for nuclei.

To determine whether GLP-1R regulated liraglutide-induced autophagy flux, we used GLP-1R siRNA to knockdown GLP-1R in PC12 cell prior to liraglutide treatment. GLP-1R siRNA knockdown efficiency is shown in Figure 8A, and further corroborated by immunofluorescence (Figure 8B). GLP-1R-siRNA markedly lowered the ratio of LC3-II/LC3-I level after liraglutide treatment, when compared with both the control group and the negative control siRNA group (Figure 8A and 8E). This indicated that GLP1-R reduced liraglutide associated autophagy induction. GLP1-R knockdown also increased ratio of p-mTOR/mTOR compared to liraglutide control (Figure 8A and 8C), showing that reduced GLP-1R increased mTOR activity after liraglutide treatment. To further demonstrate that liraglutide enhanced autophagy flux, we used the liraglutide in PC12 cells with another autphagy inhibitor, bafilomycin A1 (Baf A1), which blocked the fusion of autophagosomes with lysosomes) [29]. Immunofluorescence results of LC3 showed up-regulated expression of autophagic LC3 in liraglutide and bafilomycin A1group, compared to liraglutide and bafilomycin

A1-treated alone group. However, GLP-1R knockdown reversed this effect (Figure 8D).

### GLP-1R activation is required for liraglutide-induced autophagy and mTOR inhibition under H_2_O_2_ stimulation

**Figure 9:**
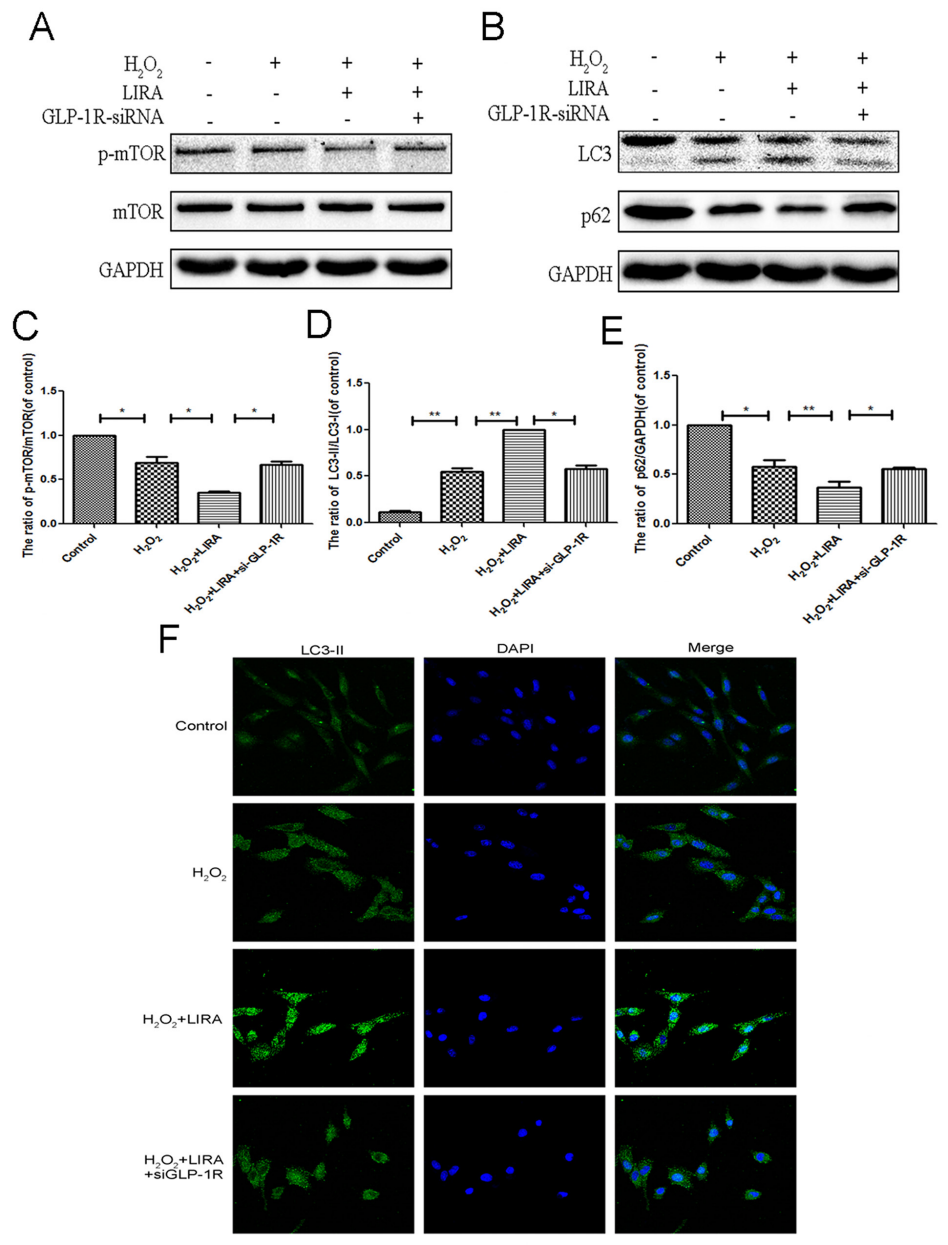
Inhibition of GLP-1R by siRNA markly attenuated liraglutide-induced autophagy and the inhibition of mTOR pathway in PC12 cells under H2O2 stimulation We planted the cells into the six-well plate, when cells came to the 60–70% confluency, we transfected siRNA. Six hours after transfection, medium was switched to medium with 5% FBS for 24 hours. Cells were pretreated with or without liraglutide for 1 h before the addition of H2O2 (100 μM) for 24 h. **(A-E)** Representative western blots and quantification data of p-mTOR, mTOR, LC3 and P62 in the PC12 cells as treated above; columns represent mean ± SD, significant differences between the treatment and control groups are indicated as **P<0.01, *P<0.05, n=5. **(F)** The LC3B (green) was detected by immunofluorescence staining combined with DAPI staining for nuclei in PC12 cells.

In order to better understand if autophagy flux was dependent on liraglutide binding to the GLP-1R, we knocked down GLP-1R in PC12 cells as discussed above. H_2_O_2_ induced autophagy as shown by increased the LC3-II/LC3-I ratio, and this was further exacerbated with liraglutide treatment. p-mTOR/mTOR ratio and p62 levels were decreased in the liraglutide treated H_2_O_2_ group. GLP-1R siRNA blocked liraglutide induced autophagic flux as shown by lowered LC3-II/LC3-I ratio and upregulated p62 compared to liraglutide H_2_O_2_ control. The radio of p-mTOR/mTOR was greatly increased in the GLP-1R knockdown group as well (Figure 9A-9E). We next used immunofluorescence to look at LC3-positive puncta, an indicator of active autophagy. Increased LC3-positive puncta were observed in the liraglutide treated H_2_O_2_ group but reduced with GLP-1R knockdown (Figure 9F). These results indicated that GLP-1R siRNA could suppress autophagy activation and activate mTOR signaling.

### GLP-1R mediates the beneficial effect of liraglutide against H_2_O_2_-induced apoptosis

**Figure 10:**
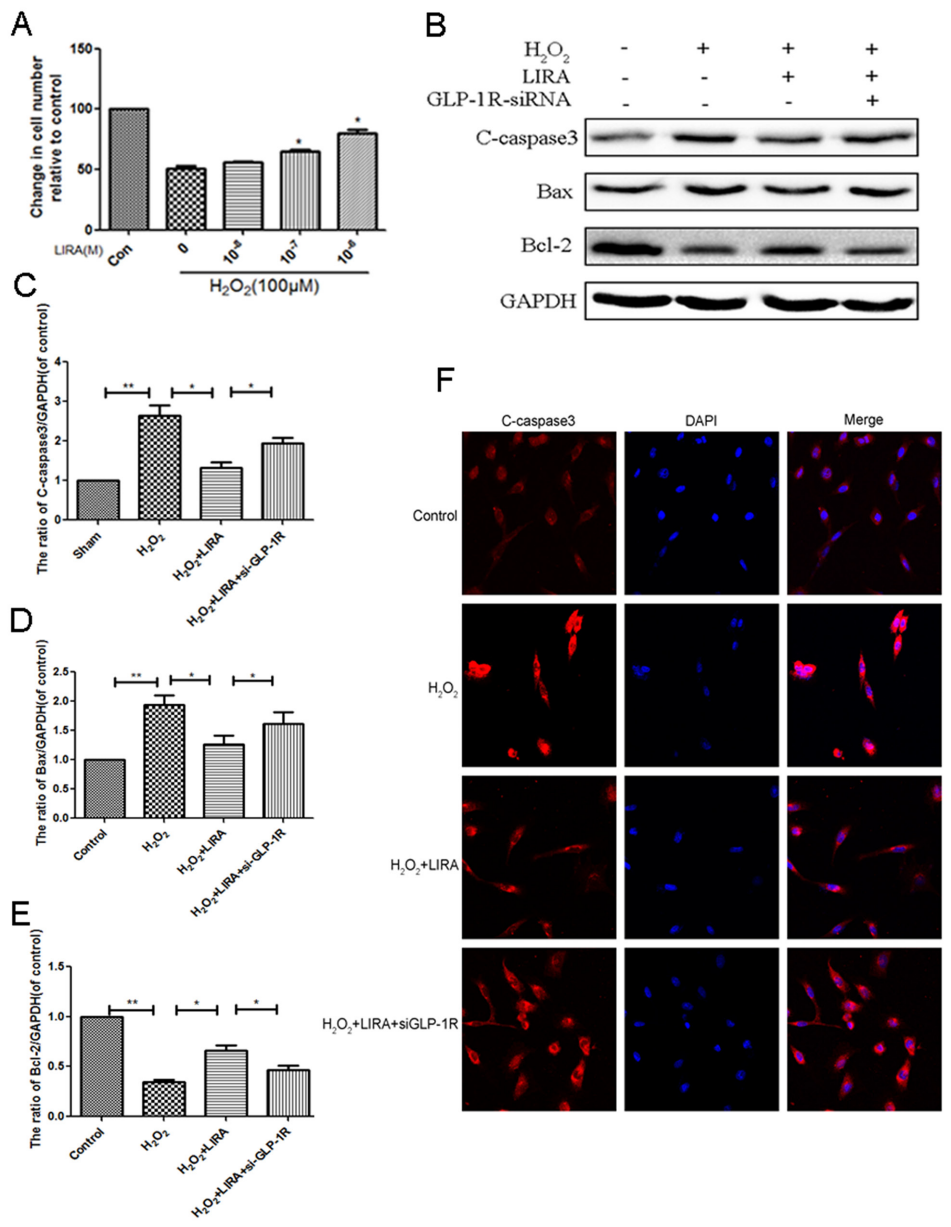
Inhibition of GLP-1R by siRNA markly attenuated the effect of liraglutide against apoptosis induced by H2O2 in PC12 cells **(A)** CCK-8 results of liraglutide-pretreated PC12 cells induced by H2O. **(B-E)** Representative western blots and quantification data of cleaved caspase 3, Bax and Bcl-2 in each group rats; columns represent mean ± SD, Significant differences between the treatment and sham groups are indicated as *P<0.05, **P<0.01, n=5. **(F)** The caspase 3 (red) was detected by immunofluorescence staining combined with DAPI staining for nuclei in PC12 cells.

Using CCK-8 assays, liraglutide improved cell viability after H_2_O_2_ treatment (Figure 10A). We investigated if the GLP-1R is mechanistically important for the increased cell viability. Cells were pretreated with GLP-1R-siRNA. Liraglutide markedly reduced levels of pro-apoptotic cleaved caspase3 and Bax, and increased the level of anti-apoptotic Bcl-2 under H_2_O_2_ stimulation. However, GLP-1R knockdown reversed liraglutide’s anti-apoptotic effect (Figure 10B-10E). Similarly, immunofluorescent staining result showed that GLP-1R knockdown increased cleaved-caspase3-positive puncta compared to the liraglutide treated H_2_O_2_ group (Figure 10F). Together, these results show that GLP-1R is necessary for the increased survival seen with liraglutide treatment.

## DISCUSSION

Trauma-induced SCI is a major cause of death and lifelong disability in the world. After crush injury, the initial injury period is followed by a longer secondary injury period including oxidative stress, inflammation, edema and apoptosis [30]. The main obstacle to recovery from the secondary injury phase is neuronal cell death. Several studies *in vivo* have demonstrated the presence of neuronal cell death and axonal interruption in acute CNS injury [31–33]. Studies including our work show that pathogenic factors such as reactive oxidative stress (ROS) can trigger apoptosis and delayed death of cells in the ischemic penumbral region due to a buildup of oxidative damage to macromolecules, such as protein and DNA oxidation [34–36]. Moreover, excess oxidative stress compromises the function of axon via accelerating the degradation of microtubules [37]. Microtubules and microfilaments are susceptible to oxidation, which inhibit the polarization of microtubules and cause the destruction of microfilaments in neurons [38]. So, the equilibrium of ROS shifts to favor ROS production after cerebral ischemia, leading to cell injury [39, 40]. Moreover, ROS contributes not only to macromolecular damage but also as signaling molecules that activate certain signal transduction pathways, including pro-apoptotic neuronal cell death signaling [41]. Bcl-2 family proteins including cytochrome c, AIF, endonuclease G, are activated and interact with each other to release the pro-apoptotic proteins, which accumulated in the mitochondrial intermembrane space. This sequence of events activates neuronal apoptosis [42–44] and is regarded as the ‘intrinsic pathway’. Other signal transduction pathways activated by ROS include the PI3K, p53, and MAPK pathways, which can all modulate intrinsic apoptosis [45–47]. Liraglutide had been mentioned to attenuate oxidative stress in various studies. GLP-1 receptor activation by liraglutidegreatly reduced nitro-oxidative stress in endotoxaemic mice [48]. And liraglutidedecreased oxidized LDL-inducedoxidative stressand fatty degeneration via regulation of AMPK signaling [49].

Increasing evidences showed that inhibiting neuronal apoptosis is an effective approach to facilitate functional and pathological recovery after CNS injury [50, 51]. Several studies have demonstrated the critical effect of GLP-1R on reducing oxidative stress and inhibiting apoptosis. Activating GLP-1R stimulates the adenylyl cyclase signaling, causing an increase of cAMP levels, which activate PKA and subsequently phosphorylate CREB. Then activation of CREB by GLP-1R regulates the transcription of the bcl-2 family protein, which plays an important role in cell growth and survival [52]. The GLP-1 analog, exendin-4, protects against ischemia-induced neuronal apoptosis by inducing GLP-1R expression in a model for transient cerebral ischemia [53]. A 2-months prospective pilot study showed that liraglutide decrease oxidative stress induced by T2DM [54]. Additionally, liraglutide promotes neuron survival and attenuates oxidative stress induced-apoptosis in the brain [55]. GLP-1R antagonist exendin-(9-39) was shown to reverse the protective effects of geniposide via upregulating the level of cleaved caspase 3 and activation of apoptosis [56]. In our study, we used Bcl-2, Bax, and cleaved caspase 3 as markers to measure apoptotic activation after SCI. Bcl-2 inhibits apoptosis, Bax is released upon initiation of apoptotic process, and cleaved caspase 3 mediates cleavage of cellular components [57, 58]. Our results showed that liraglutide treatment markedly reduced expression of pro-apoptotic Bax and cleaved caspase3, and the decreased anti-apoptotic Bcl-2 in rats after SCI. We also demonstrated that the GLP-1R is mainly expressed in neurons rather than nervous system cell types in spinal cord tissue. GLP-1R expression increases after acute SCI, indicating that it may affect neuronal survival. To explore whether GLP-1R is neuroprotective, we knocked down GLP-1R in neuronal cells *in vitro*. GLP-1R siRNA reversed liraglutide’s anti-apoptotic effect with H_2_O_2_ stimulation, indicating that the GLP-1R was necessary to reduce apoptosis with liraglutide treatment after SCI.

Several articles have reported an interaction between autophagy and microtubule stability [8, 16]. However, the mechanisms and reasoning for this remain poorly understood. Autophagy maintains cellular homeostasis by degrading cytoplasmic components including organelles or protein aggregates [59]. Increased autophagy has been reported after SCI as a self-protective mechanism in response to various injury-associated pathological factors such as energy deficiency and oxidative stress [60, 61]. In addition, many retraction bulbs, which are associated with disorganized microtubule structure, have been found in injured CNS axons [62].

Stable microtubular structures such as axonemes have been shown to contain acetylated alpha-tubulin with acetylation occuring after microtubule assembly [28]. Increasing microtubule acetylation repairs locomotor deficits and axonal transport caused by LRRK2 Roc-COR domain mutations [27]. Additionally, resveratrol inhibits axonal degeneration in Wallerian degeneration mice by activating SIRT2 (NAD-dependent tubulin deacetylase) [63]. Exendin-4 induces extracellular superoxide dismutase to combat oxidative stress through acetylation of histone H3 [64]. Interestingly, it was reported that autophagy is activated in both the early and duration of degenerating axons after SCI [65], indicating that autophagy could potentially represent a therapeutic target to reduce axonal degeneration in CNS injury. Autophagy induction was shown to stabilize microtubules by degradading microtubule destabilizing protein in cultured CNS neurons as well as promoting axon growth. Furthermore, activation of autophagy by a specific autophagy-inducing peptide, Tat-beclin1, could promote microtubule polymerization, axon regeneration, and locomotor functional recovery in mice after SCI [8]. In this study, we found that liraglutide increased autophagic flux and microtubule acetylation in rats after SCI. Furthermore, we blocked the downstream of autophagy by bafilomycin A1 in PC12 cells, and the expression of LC3 was further up-regulated in liraglutide-treated cells [66]. However, siGLP-1R abolished this effect, which further indicated that liraglutide enhanced autophagy flux via activating GLP-1R. Moreover, autophagy inhibition with 3-MA (type III PI3 kinase inhibitor, which inhibits autophagosome formation) reduced microtubule acetylation *in vivo*, indicating autophagy was important for microtubule stability.

GLP-1 analogs have been reported in the literature several times to exert neuroprotective effects via activation of autophagy [26]. However, it remains unclear whether liraglutide activates autophagy by binding to the GLP-1R. Beta-glycerophosphate -induced increase of p-mTOR^Ser2448^, and p-S6K1^Thr389^ were attenuated by liraglutide treatment of human vascular smooth muscle cells [67]. Inhibition of mTOR signaling is a classic autophagy activation pathway [68]. mTOR inhibition initiates autophagy through activation of type III PI3 kinase, which is necessary for autophagosome biogenesis. In addition, mTOR inhibition can activates transcription of lysosomal genes, which stimulate lysosomal biogenesis [69]. Previous reports have suggested that inhibiting the mTOR pathway could be neuroprotective by enhancing autophagy after CNS injury [70, 71].

**Figure 11:**
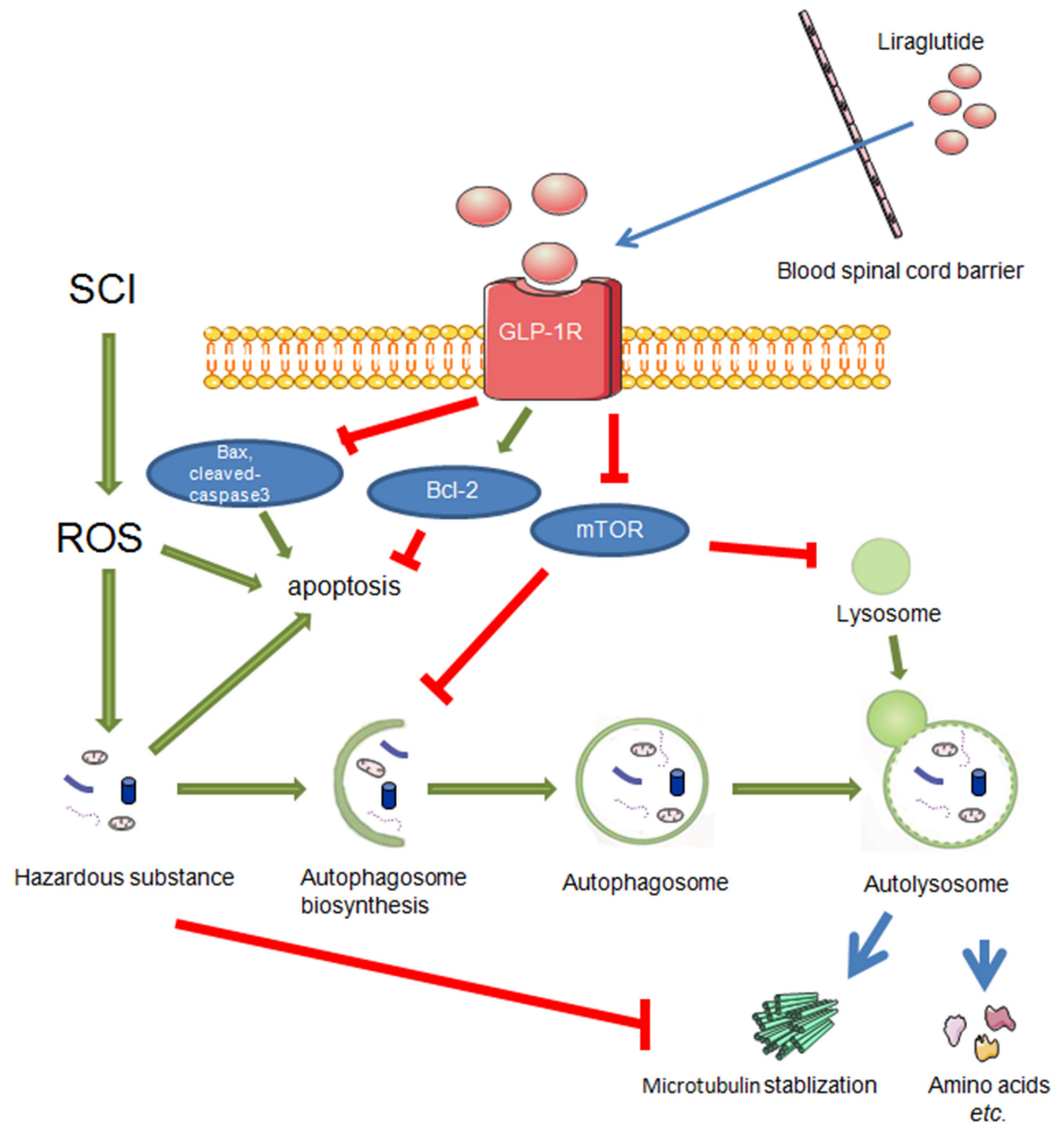
During the secondary injury of SCI, ROS mainly results in accumulation of hazardous substance which eventually brings about apoptosis On the other hand, liraglutde treatment promotes autophagy flux and decrease apoptosis via GLP-1R. And autophagy stabilized microtubules by degrading a microtubule destabilizing protein.

In this study, we found that GLP-1R knockdown in neuronal cells *in vitro* significantly reduced liraglutide-induced autophagy. Liraglutide treatment inhibited mTOR activation, which was also reversed by GLP-1R knockdown. Moreover, GLP-1R siRNA prevented increased autophagic flux associated with liraglutide treatment. The ratio of p-mTOR/mTOR was increased in neuronal cultures under H_2_O_2_ stimulation as well, indicated that GLP-1R knockdown could activate mTOR signaling and suppress autophagy activation (Figure 11).

In summary, this is the first study demonstrating that liragutide facilitates better functional recovery and increased microtubule acetylation/polymerization concurrently in rats after SCI. Liraglutide’s beneficial effects in the recovery process are mediated by autophagy induction and mTOR suppression. Importantly, the GLP-1R is essential for activation of autophagy with liraglutide treatment.. Liraglutide prevents SCI induced apoptosis in rats, but GLP-1R knockdown with liraglutide treatment abolished this anti-apoptotic effect in neuronal cultures. Thus, GLP-1R may play a key role in physiological recovery after CNS injury. Mechanistically, GLP-1R activation can subsequently activate autophagy flux, which is involved in both axon regeneration and microtubule stabilization preventing further damage after injury.

## MATERIALS AND METHODS

### Reagents and antibodies

Clinical-grade liraglutide was obtained from Novo Nordisk (Princeton, NJ, USA). Antibodies against GLP-1R, Bax and Bcl-2 were obtained from Santa Cruz Biotechnology (Santa Cruz, CA, USA). Antibodies against LC3, LC3-II and acetyl-α-tubulin were acquired from Cell Signaling Technologies (Danvers, MA, USA). Antibodies against P62, beclin-1, p-mTOR, mTOR, and caspase3 were purchased from Abcam (Cambridge, MA, USA). All other reagents were obtained from Sigma-Aldrich (St. Louis, MO, USA) unless specified otherwise.

### Spinal cord injury

All the adult female SD rats (220–250 g) were obtained from the Animal Center of the Chinese Academy of Sciences in Shanghai, China. All surgical interventions, treatments, and postoperative animal care procedures were performed in strict accordance with the Animal Care and Use Committee of Wenzhou Medical University. All rats were housed in the SPF Laboratory Animal Room. After anesthesia by chloralic hydras (3.6 ml/kg, i.p.), rats were positioned on an operating table. After an incision on the midline of the back at the T9 vertebrae, a laminectomy was performed. The spinal cord was compressed by vascular clamp (30 g force; Oscar, China) for 1 min to moderate crushing injury. And sham group only received a T9 laminectomy. After operation, we emptied urinary bladder every 12 hrs until the recovery of normal bladder function and cefazolin sodium (50 mg/kg) was intraperitoneally administrated in the first three days. Liraglutide was dissolved in saline and administered at 50 ug/kg subcutaneously (s.c.) near the back wound until the rats were killed [55]. After surgery, another group of rats was injected with liraglutide (50 ug/kg/day) and an autophagy inhibitor 3-methyladenine (3-MA, 200 nmol/kg, i.p.) [72]. Equal saline were administrated for the sham group.

### Cell culture and drug treatment

PC12 cells were purchased from the Cell Bank of Type Culture Collection of Chinese Academy of Sciences, Shanghai Institute of Cell Biology, Chinese Academy of Sciences. They were cultured in RPMI 1640 medium with 10 % FBS, 100U/ml penicillin, and 100U/ml streptomycin in a cell incubator containing 5 % CO2. Cells were pretreated with concurrent concentration-dependent treatment of liraglutide for 1 h before the addition of H2O2 (100 μM) for 24 h for cell viability experiments. All experiments were performed at least three times.

### Locomotion recovery assessment

The Basso, Beattie, and Bresnahan (BBB) scoring system was used to assess the locomotion recovery in rats with SCI [70], which range from 21 points (normal locomotion) to 0 points (complete paralysis). In this study, two blinded independent researchers measured the BBB scores at 1, 3, 7, 14, 21, and 28 days after surgery.

### Hematoxylin–eosin (HE) staining and nissl staining

To measure the cavity area of spinal cord tissue in each group after SCI, rats in all groups were sacrificed 4 weeks after surgery. Spinal cord tissue was cut into serial longitudinal sections of 5 μm thickness for HE staining. To measure the surviving neurons in the spinal cord tissue of each group, tissue segments (1 cm on each side of the lesion) were embedded in paraffin and transverse paraffin sections incubated in 1 % cresyl violet acetate solution for Nissl staining. We counted the number of ventral motor neuron (VMN) as shown in previous study [73].

### Transmission electron microscopy

Rats were killed at 3 days after surgery. Following fixation in 2.5% glutaraldehyde and 2% osmium tetroxide, spinal cord tissues (4–5mm caudal and rostral) were blocked with 2% uranyl acetate. Dehydrated by acetone washes, tissues were embedded in Araldite for Semi-thin sectioning to determine the location. At last, ultra-thin sections of each sample were cut for observation. All images were captured using a Hitachi TEM.

### The terminal deoxynucleotidyl transferase (TdT) dUTP nick end labeling (TUNEL) method

To measure apoptotic level, sections (4–5mm caudal and rostral) were obtained and TUNEL staining was performed at 7 days after SCI. Deparaffinized in xylene and rehydrated by ethanol washing, sections were incubated with 0.1% Triton X-100 for 30 min. The apoptotic cells of spinal cord were stained with In Situ Cell Death Detection Kit (Roche Molecular Biochemicals) and 40, 6-diamidino-2-phenylindole (DAPI). All images were captured using a Nikon ECLIPSE Ti microscope (Nikon, Japan). And TUNEL-positive cells were counted from 20-30 random sections to get an average number in each group, with three rats measured in each group.

### Western blot analysis

T7 to T10 level of spinal cord tissue were obtained at 3 days and 28 days after surgery. Briefly, spinal cord tissues and cells were isolated using RIPA buffer containing phosphatase and protease inhibitors. Protein concentration was measured using bicinchoninic acid reagents (Thermo, Rockford, IL, USA). Total proteins were separated with 8–12% SDS–PAGE gels and transferred onto polyvinylidene fluoride membranes (Bio-Rad, CA, USA). The membranes were blocked with 5% skim milk, incubated with primary antibodies, and then respective secondary antibodies. Signals were measured and quantified by the ChemiDicTM XRS + Imaging System (Bio-Rad). Experiments were repeated three times.

### Immunofluorescence staining

At 3 days and 4 weeks after surgery, spinal cord tissue was embedded in paraffin. Transverse and longitudinal sections (5 μm thick) were cut, deparaffinized in xylene, and rehydrated by ethanol washing. And cells cultured on 18 × 18 mm microscopic glasses and fixed in 4 % paraformaldehyde. Then tissues and cells were incubated by 5% BSA for 1 hr, the following primary antibodies were incubated: anti-LC3B (1:500), anti- acetyl-α-tubulin (1:1000 Technology), GLP-1R (1:200) caspase3 (1:500), anti-NeuN (1:1000), anti-GFAP (1:1000), and anti-Iba-1 (1:500), followed by incubation with respective secondary antibodies for 1 h and DAPI for 7 min. All images were captured using a Nikon ECLIPSE Ti microscope (Nikon, Japan).

### Small interfering RNA (siRNA)

GLP-1R siRNA were used to knockdown the expression of GLP-1R. PC12 cells were transfected with 100 pmol of GLP-1R siRNA (GeneChem, Shanghai, China) with lipofectamine 2000 (Life Technologies, Carlsbad, CA, USA) according to the instructions of manufacturer. Six hours after transfection, medium was switched to medium with 5% FBS for 24 hours.

### Statistical analysis

The results are presented as the mean ± SD from three independent experiments. Statistical analyses were performed using Graphpad Prism (USA) ( one-way analysis of variance (ANOVA) followed by Tukey’s test). P <0.05 was considered statistically significant.
